# Hairpin‐Spacer crRNA‐Enhanced CRISPR/Cas13a System Promotes the Specificity of Single Nucleotide Polymorphism (SNP) Identification

**DOI:** 10.1002/advs.202003611

**Published:** 2021-01-31

**Authors:** Yuqing Ke, Shiyi Huang, Behafarid Ghalandari, Sijie Li, Antony R. Warden, Jingqi Dang, Lin Kang, Yu Zhang, Yunqing Wang, Yiqing Sun, Jinglin Wang, Daxiang Cui, Xiao Zhi, Xianting Ding

**Affiliations:** ^1^ State Key Laboratory of Oncogenes and Related Genes Institute for Personalized Medicine School of Biomedical Engineering Shanghai Jiao Tong University Shanghai 200030 China; ^2^ State Key Laboratory of Pathogen and Biosecurity Institute of Microbiology and Epidemiology Beijing 100071 China; ^3^ Shanghai Engineering Centre for Intelligent Diagnosis and Treatment Instrument School of Electronic Information and Electrical Engineering Shanghai Jiao Tong University Shanghai 200240 China

**Keywords:** CRISPR/Cas13a system, crRNA design, Gibbs free energy, hairpin structures, single nucleotide polymorphisms

## Abstract

The Cas13a system has great potential in RNA interference and molecular diagnostic fields. However, lacking guidelines for crRNA design hinders practical applications of the Cas13a system in RNA editing and single nucleotide polymorphism identification. This study posits that crRNAs with hairpin spacers improve the specificity of CRISPR/Cas13a system (termed hs‐CRISPR). Gibbs free energy analysis suggests that the hairpin‐spacer crRNAs (hs‐crRNAs) suppress Cas13a's affinity to off‐target RNA. A hepatitis B virus DNA genotyping platform is established to further validate the high‐specificity of hs‐CRISPR/Cas13a system. Compared to ordinary crRNA, hs‐crRNAs increase the specificity by threefold without sacrificing the sensitivity of the CRISPR/Cas13a system. Furthermore, the mechanism of the Cas13a/hs‐crRNA/target RNA composition is elucidated with theoretical simulations. This work builds on the fundamental understanding of Cas13a activation and offers significant improvements for the rational design of crRNA for the CRISPR/Cas13a system.

## Graphical abstract

This study proposes that hairpin‐spacer crRNAs (hs‐crRNA) improve the specificity of CRISPR/Cas13a system (hs‐CRISPR). Gibbs free energy analysis and experimental data demonstrate that hs‐crRNAs suppress Cas13a's activity to nonspecific targets. This work provides a fundamental understanding of Cas13a activation associated with binding energetics and an efficient strategy for high‐specificity crRNA design.

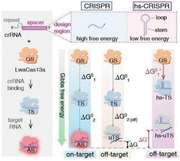

## 1 Introduction

Clustered regularly interspaced short palindromic repeats (CRISPR) and CRISPR‐associated (Cas) protein systems offer various possibilities in molecular diagnostics^[^ ^1^, ^2^, ^3^ ^]^ and RNA interference^[^ ^4^, ^5^, ^6^ ^]^ beyond genome editing^[^ ^7^, ^8^ ^]^ due to the programmability of nuclease activity. The activity of CRISPR/Cas13a, an RNA‐guided RNase, is determined by higher eukaryotes and prokaryotes nucleotide‐binding (HEPN) domains.^[^ ^9^, ^10^ ^]^ Cas13a can combine with CRISPR RNA (crRNA) to form the Cas13a/crRNA duplex, which is competent at recognizing target RNA with the guidance of crRNA‐spacer. Once assembled with target RNA, the Cas13a/crRNA/target RNA ternary complex will activate the HEPN domains to create a general RNase active site in the Cas13a protein.^[^ ^11^, ^12^ ^]^ These properties impart CRISPR/Cas13a with advantages of rapid, accurate, high‐sensitivity, and programmability for molecular diagnostics.^[^ ^13^, ^14^, ^15^ ^]^ However, CRISPR/Cas systems are still challenged by high off‐target rates that affect the accuracy and stability of practical applications.^[^ ^5^, ^16^, ^17^ ^]^

Optimizing Cas proteins^[^ ^18^, ^19^ ^]^ and/or crRNA design^[^ ^20^, ^21^, ^22^ ^]^ are prominent strategies that increase efficacy. The clarification of activation mechanisms via probing structures helps the development of high‐performance Cas13a proteins.^[^ ^9^, ^23^, ^24^ ^]^ The most common method to optimize crRNA is to introduce synthetic mismatch into the crRNA‐spacer, which promotes the identification ability of single nucleotide polymorphisms (SNPs) toward Cas13a proteins.^[^ ^15^, ^20^, ^21^, ^25^ ^]^ However, Cas13a protein optimization remains challenging due to its sophisticated nature. Besides, the location of synthetic mismatch in crRNA is uncertain and its enhancing mechanism is unclear. In recent years, studies show that hairpin structures enhance the functions of RNA molecules in extensive applications, such as strand‐displacement amplification,^[^ ^26^ ^]^ RNA interference,^[^ ^27^ ^]^ aptamer biosensing,^[^ ^28^, ^29^, ^30^ ^]^ and RNA origami.^[^ ^31^, ^32^ ^]^ Furthermore, RNA secondary structures increase the specificity of Cas9 and Cas12a systems in genetic engineering.^[^ ^33^ ^]^ However, little is known regarding the value of hairpin‐spacer structures in the CRISPR/Cas13a system while applied to molecular diagnostics and RNA interference.

**Figure 1:**
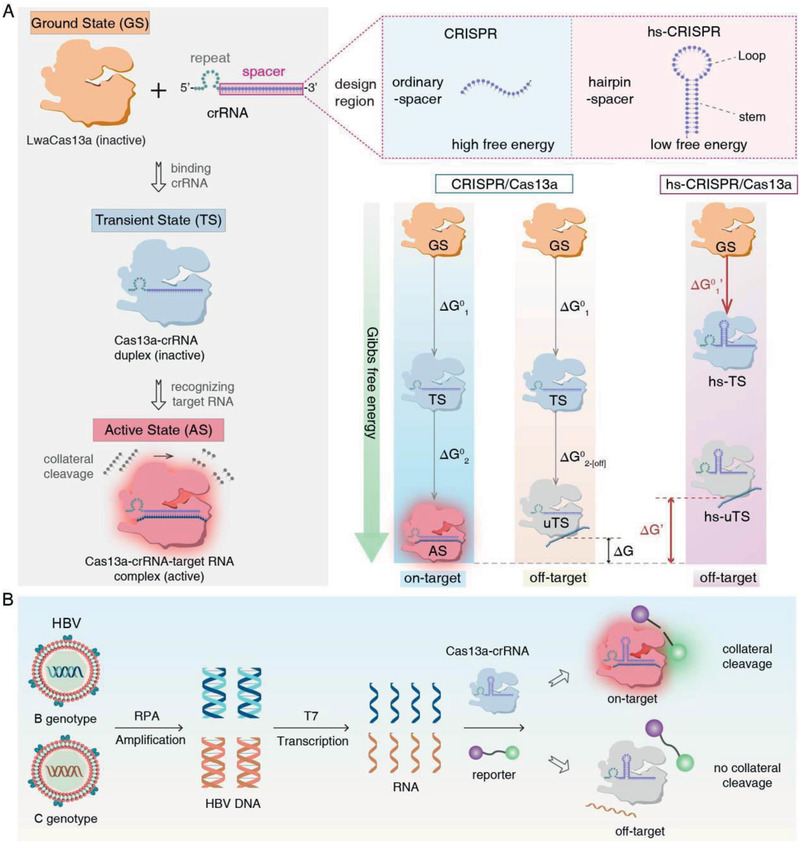
Overview of hs‐CRISPR method. A) Schematic detailing the process of Cas13a nuclease activation through crRNA binding and target RNA recognition. Cas13a starts with the ground state (GS), then binds with crRNA and steps into the transient state (TS). Cas13a in TS can recognize specific target RNA guided by crRNA and shows nuclease activity, entering the active state (AS). crRNA consists of direct repeat (dot in green) and spacer (dot in purple) structures. We designed three crRNAs with or without hairpin spacer. Basic hairpins are composed of stems and loops. The design parameters include location and length of stems. The Gibbs free energy changes from GS to TS (termed “Δ*G* ^0^_1_”) and TS to AS (termed “Δ*G* ^0^_2_”) represent the binding affinity of Cas13a to crRNA and target RNA, respectively. Δ*G* ^0^_1_ for the hs‐CRISPR/Cas13a system is labeled as Δ*G* ^0^_1_′. The difference in binding affinity of Cas13a to on‐target and off‐target RNA was labeled as “Δ*G*”. Δ*G* for the hs‐CRISPR/Cas13a system is labeled as Δ*G*′. B) Workflow of HBV genotyping platform based on hs‐CRISPR. HBV DNA is amplified by RPA reaction, then transcribed into RNA, which can be recognized by Cas13a/crRNA duplex. Notably, due to the nucleotide polymorphisms caused by genotypes, collateral cleavage will not occur for off‐target RNA.

Herein, we propose a hairpin‐spacer crRNA (hs‐crRNA) strategy that improves the specificity of the CRISPR/Cas13a system (hs‐CRISPR) by suppressing the binding affinity between Cas13a and off‐target RNA (**Figure** **1**A). First, we investigated the enhancing mechanism of hs‐CRISPR and analyzed the variation of Gibbs free energy between the interaction of Cas13a with hairpin‐spacer crRNA and ordinary crRNA. Next, we evaluated the practical efficacy of hs‐CRISPR at genotyping hepatitis B virus (HBV) DNA (Figure 1B). Last, we examine the structural information of the Cas13a/hs‐crRNA duplex and Cas13a/hs‐crRNA/target‐RNA complex via simulation.

## 2 Results and Discussion

### 2.1 Assay Scheme of hs‐CRISPR

The principle of hs‐CRISPR is described in Figure 1. The main basis of this system is the target RNA recognition of Cas13a/crRNA duplex and the resulting collateral *trans*‐cleavage (Figure 1A). At first, Cas13a is in the ground state (GS) and shows no nuclease activity. Then it can bind with crRNA and step into the transient state (TS). Cas13a/crRNA duplex in TS can be guided by crRNA‐spacer to recognize specific target RNA and shows RNase activity, being in the active state (AS). While Cas13a/crRNA interacts with off‐target RNA, Cas13a will be in undesired transient state (uTS) with no nuclease activity. To investigate the effects of hairpin crRNA‐spacer structures on Cas13a activity, we designed crRNAs with or without engineered hairpin‐spacer. Hairpin structures were created by extending the 3′‐flank of crRNA with several bases complementary to spacer sequence. Basic hairpins are composed of stems and loops. By tuning the location and length of stems, we obtained different hairpin‐spacer crRNAs (hs‐crRNAs). Furthermore, we measured the Gibbs free energy changes from GS to TS (termed “Δ*G* ^0^_1_”) and TS to AS (“Δ*G* ^0^_2‐[on]_”) or TS to uTS (termed “Δ*G* ^0^_2‐[off]_”) using fluorescence spectrometer. The difference in Δ*G* ^0^_2‐[on]_ and Δ*G* ^0^_2‐[off]_ was determined as “Δ*G*”. For hs‐CRISPR, crRNA with low free energy was used and therefore high Δ*G* was obtained, making off‐target RNA unable to activate the nuclease activity of Cas13a.

To verify the advanced discrimination ability of hs‐CRISPR on SNPs, we further compared hs‐crRNAs with ordinary crRNA (o‐crRNA) for HBV genotyping (Figure 1B). Considering HBV genotypes B and C are major subpopulations in Oceania and Asia, we selected HBV B‐type and C‐type as our genotyping targets for proof‐of‐concept in this study. HBV DNA are amplified by recombinase polymerase amplification (RPA) reaction. Then amplified products are transcribed into related RNAs using in vitro T7 transcription system, which function as targets of Cas13a/crRNA duplex. Notably, owing to the nucleotide polymorphism caused by different genotypes, HBV C‐type cannot activate the RNase activity of LwaCas13a binding with HBV B‐type crRNA (B crRNA).

### 2.2 Hs‐crRNAs Show Lower Affinity to Cas13a than Ordinary crRNA

**Figure 2:**
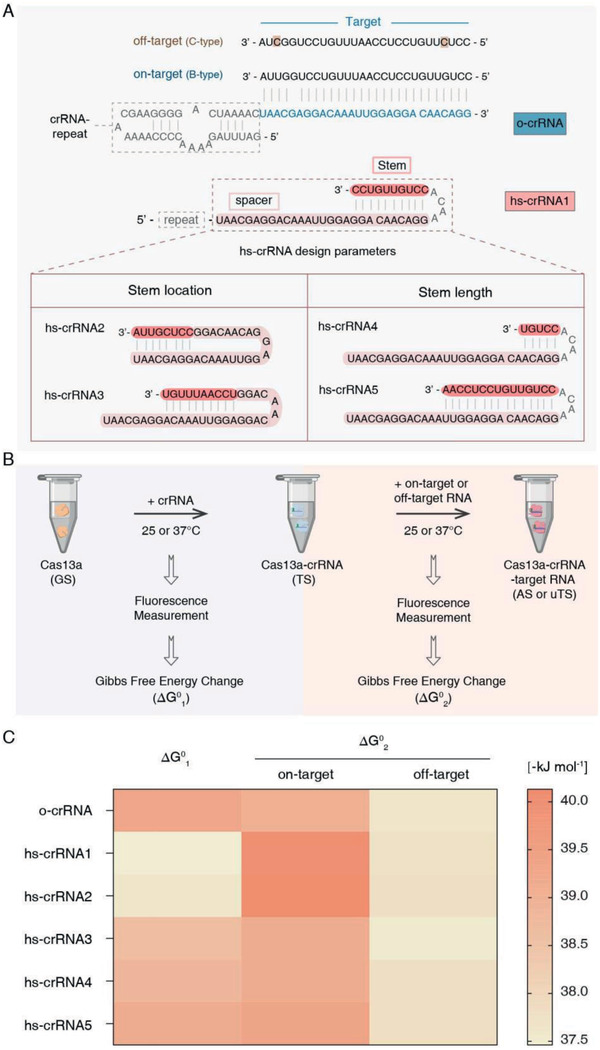
Hs‐crRNAs suppress the Cas13a's overall affinity to off‐target RNA. A) crRNA‐spacers design of ordinary crRNA (o‐crRNA) and hs‐crRNAs. SNP‐induced mismatches are highlighted in brown for HBV C‐type DNA (off‐target). The crRNA‐repeat region is marked in gray. The design parameters of hs‐crRNA spacers include the stem location and length. The stem and spacer structures of hs‐crRNAs are highlighted in orange and pink, respectively. The stems of hs‐crRNA2 and hs‐crRNA3 are located at different position in the spacer, while hs‐crRNA4 and hs‐crRNA5 possess 5‐bp and 15‐bp stems, respectively. B) Measurements of intrinsic fluorescence spectra of Cas13a protein with crRNAs and target RNA at 25 and 37 °C. Cas13a was first incubated with crRNA (0 × 10^−6^–2.5 × 10^−6^ m), then Cas13a/crRNA duplex was incubated with target RNA (concentrations ranging from 0 × 10^−6^ to 1 × 10^−6^ m) and the spectra were obtained by fluorescence spectrometer. Fluorescence spectra were further analyzed using Stern–Volmer equations to calculate the Gibbs free energy chages. C) Calculated Gibbs free energy changes of Cas13a interacting with crRNA (termed “Δ*G* ^0^_1_”) and on‐target (termed “Δ*G* ^0^_2‐[on]_”) or off‐target RNA (termed “Δ*G* ^0^_2‐[off]_”) at 37 °C for o‐crRNA and hs‐crRNAs.

To explore the effects of hairpin crRNA‐spacers on Cas13a activity, we compared the capabilities of crRNAs with or without hairpin spacer for HBV DNA genotyping. As shown in **Figure** **2**A, a 28‐nt sequence of HBV containing two SNPs was chosen as the target of o‐crRNA. By extending the 3′‐end of o‐crRNA to form a 10‐bp stem structure at the distal region to crRNA‐repeat, we obtained hs‐crRNA1. By changing the stem location and length of hairpin structure in crRNA‐spacer, we further designed four different hs‐crRNAs, named hs‐crRNA2 to hs‐crRNA5, respectively. The stem structures of hs‐crRNA2 located at the proximal region to crRNA‐repeat while that of hs‐crRNA3 showed an 8‐nt distance from crRNA‐repeat. The stem lengths of hs‐crRNA4 and hs‐crRNA5 were 5‐bp and 15‐bp, respectively. The sequences of all crRNAs are supplied in Table S1 of the Supporting Information. Furthermore, we predicted the minimum free energy structures of crRNA‐spacers using RNAstructure online tools and RNAfold webserver (Figure S1, Supporting Information). We discovered that hs‐crRNAs had lower predicted minimum free energies (−68.20, −74.48, −43.5136, −23.012, and −111.7128 kJ mol^−1^ for hs‐crRNA1 to hs‐crRNA5, respectively) than o‐crRNA (−3.68 kJ mol^−1^).

To clarify the mechanism of Cas13a binding with crRNA, we investigated the conformational changes of Cas13a by fluorescence spectroscopy which has been widely applied in protein dynamics, assembly and interaction researches.^[^ ^34^, ^35^, ^36^ ^]^ The Cas13a protein has natural fluorophores (tryptophan and tyrosine residues), allowing fluorescence analysis of conformational changes. First, we incubated Cas13a (GS) with different concentrations of crRNA ranging from 0 × 10^−6^ to 2.5 × 10^−6^ m at 25 and 37 °C for 10 min, respectively (Figure 2B). Then we measured the fluorescence intensity of Cas13a/crRNA duplex (TS) and analyzed the fluorescence quenching data using the Stern–Volmer equation (Equation 1)

(1) $$\frac{F_{0}}{F}\;=\;1\;+K_{\mathrm{SV}}\left[Q\right]\;=\;1\;+k_{q}\tau_{0}\left[Q\right]$$

where *F*_0_ and *F* are the fluorescence intensity of Cas13a protein in the absence and presence of RNA, respectively. *K*_SV_ is the Stern–Volmer quenching constant, [*Q*] is the concentration of RNA, *k*_q_ is biomolecular quenching rate constant, and *τ*_0_ is the average lifetime of excited fluorophore in the absence of quencher (average *τ*_0_ = 10^−8^ s).^[^ ^37^ ^]^ A modified Stern–Volmer equation was used to calculate the exact parameters of interaction (Equation 2)

(2) $$\frac{F_{0}}{\Delta F}=\;\frac{F_{0}}{F_{0}-F}=\;\frac{1}{f_{a}K_{\mathrm{SV}}}\;\frac{1}{\left[Q\right]}+\;\frac{1}{f_{a}}$$

in which *f*_a_ is the mole fraction of accessible fluorescence and *K*_SV_ is the Stern–Volmer quenching constant of accessible fluorophores. This equation can be applied to determine the percentage of quencher accessible fluorophores.^[^ ^38^ ^]^

The fluorescence spectra of Cas13a incubating with crRNAs (0 × 10^−6^–2.5 × 10^−6^) and the Stern–Volmer plots are displayed in Figures S2 and S3 of the Supporting Information, respectively. To investigate the types of interactions between Cas13a and crRNA, we further calculated the entropy (Δ*S* ^0^) and enthalpy (Δ*H* ^0^) of Cas13a/crRNA duplexes using Equation (3)

(3) $$\log\left[\frac{F_{0}-F}{F}\right]=\;\log K_{a}+n\;\log\left[Q\right]$$

in which *K*_a_ and *n* mean the binding constant and the number of binding sites, respectively.^[^ ^39^ ^]^ Assuming unchanged entropy (Δ*S* ^0^) and enthalpy (Δ*H* ^0^) at different temperatures, the type of interactions between Cas13a protein and crRNAs or HBV RNA can be investigated using Van ’t Hoff equation (Equation 4)

(4) $$\ln K_{a}=\;-\;\frac{\Delta H^{0}}{RT}+\frac{\Delta S^{0}}{R}$$

where *R* and *T* are gas constant and absolute temperature, respectively. Changes of Standard Gibbs free energy *ΔG* ^0^_1_ were calculated using Equation (5)

(5) $$\Delta G^{0}=\;\Delta H^{0}-T\Delta \;S^{0}=\;-RT\ln K_{a}$$

According to the thermodynamic results provided in Figure 2C and Table S2 (Supporting Information), the Δ*G* ^0^_1_ value of o‐crRNA (−39.31 kJ mol^−1^) was lower than hs‐crRNAs (−37.46, −37.64, −38.64, −38.83, and −39.12 kJ mol^−1^ for hs‐crRNA1 to hs‐crRNA5, respectively) at 37 °C, demonstrating that ordinary crRNA possessed higher binding affinity toward Cas13a protein than hairpin‐spacer crRNAs. We believe that this phenomenon is caused by the RNA structure differences, since the molecular structure is one of the most critical factors to determine the affinity. It should be noted that both the structures of RNA molecules and the amounts of Cas13a's space occupied by crRNA would affect the affinity of the protein. To further investigate the effects of Cas13a's lower binding affinity on Cas13a/crRNA duplex formation, we incubated the Cas13a with o‐crRNA and hs‐crRNA1 for different time (0–30 min) and measured the fluorescence spectra of the duplex. As shown in Figure S4 of the Supporting Information, longer incubation time would not affect the fluorescence spectra of the duplex, indicating that both o‐crRNA and hs‐crRNA1 interacted with the Cas13a and formed the Cas13a/crRNA duplex spontaneously. Notably, the shapes of fluorescence spectra of the Cas13a/o‐crRNA and Cas13a/hs‐crRNA1 duplexes were different, suggesting that certain areas of Cas13a were occupied by o‐crRNA and hs‐crRNA, which may also influence the Cas13a's binding affinity to RNA.

### 2.3 Hs‐crRNAs Suppress the Cas13a's Overall Affinity to Off‐Target RNA

To investigate the interactions between Cas13a/crRNA duplex and target RNA, we further incubated Cas13a/crRNA duplex with various concentrations of on‐target or off‐target RNA (0 × 10^−6^–1 × 10^−6^ m) (Figure 2B). Similar to analyzing the interaction between Cas13a and crRNA, we recorded the fluorescence intensity of Cas13a/crRNA/target RNA complex (AS) and analyzed the fluorescence quenching data by Stern–Volmer equation. The Stern–Volmer plots of Cas13a/crRNA duplex interacting with target RNA (0 × 10^−6^ –1 × 10^−6^ m) at 25 and 37 °C are shown in Figure S5 of the Supporting Information.

Standard Gibbs free energy change Δ*G* ^0^_2_ represents the binding affinity of Cas13a/crRNA duplex to on‐target or off‐target RNA. Δ*G* ^0^_2‐[on]_ means the Δ*G* ^0^_2_ value of Cas13a/crRNA interacting with on‐target RNA, whereas Δ*G* ^0^_2‐[off]_ represents off‐target RNA. As shown in Figure 2C and Table S3 (Supporting Information), Δ*G* ^0^_2‐[off]_ values were similar for all crRNAs including o‐crRNA and hs‐crRNAs at 37 °C (−37.64, −37.74, −37.82, −37.52, −37.82, and −37.82 kJ mol^−1^ for o‐crRNA, hs‐crRNA1 to hs‐crRNA5, respectively). Whereas Δ*G* ^0^_2‐[on]_ values of hs‐crRNAs (−40.02, −40.13, −39.12, −39.13, and −39.36 kJ mol^−1^ for hs‐crRNA1 to hs‐crRNA5, respectively) were lower than o‐crRNA (−39.06 kJ mol^−1^), suggesting that hairpin‐spacer crRNAs increased the binding affinity of Cas13a/hs‐crRNA duplex to on‐target RNA. Accordingly, we attributed the improved binding affinity to optimal conformation of Cas13a induced by hairpin‐spacer structures of hs‐crRNAs.

Based on the Gibbs free energy analysis, we summarized the thermodynamic results of Δ*G* ^0^_1_ and Δ*G* ^0^_2_ in **Table** **1**. We observed that the sum of Δ*G* ^0^_1_ and Δ*G* ^0^_2‐[on]_ values for all crRNAs (<−77.48 kJ mol^−1^) were lower than that of Δ*G* ^0^_1_ and Δ*G* ^0^_2‐[off]_ values (> −76.95 kJ mol^−1^), indicating that the Cas13a protein possessed an overall high‐level affinity to on‐target RNA to be in the active state (AS). Notably, the difference of Cas13a's binding affinity to on‐target and off‐target RNA was determined as “Δ*G*” (Δ*G* = Δ*G* ^0^_2‐[off]_ – Δ*G* ^0^_2‐[on]_). As shown in Table 1, hs‐crRNAs showed an increased Δ*G* values (2.38, 2.31, 1.6, and 1.54 kJ mol^−1^ for hs‐crRNA1, hs‐crRNA2, hs‐crRNA3, and hs‐crRNA5, respectively) compared to o‐crRNA (1.42 kJ mol^−1^). This observation indicated that hs‐crRNAs suppressed the Cas13a's overall affinity to off‐target RNA, making the Cas13a/hs‐crRNA/off‐target RNA complex unable to be in the AS due to the insufficient Gibbs free energy changes. Based on our thermodynamic results, we hypothesized that Δ*G* values may be associated with the specificity of Cas13a system. Since higher Δ*G* values were obtained using hs‐crRNAs compared to o‐crRNA, we believed that it was determined by the hairpin structures which may affect Cas13a's conformational changes during crRNA binding and target RNA recognition.

**Table 1:** Thermodynamic results of the Cas13a protein interacting with crRNA and target RNA at 37 °C

Group	Δ*G* ^0^_1_ + Δ*G* ^0^_2‐[on]_ [kJ mol^−1^]	Δ*G* ^0^_1_ + Δ*G* ^0^_2‐[off]_ [kJ mol^−1^]	Δ*G* [kJ mol^−1^]
o‐crRNA	−78.37	−76.95	1.42
hs‐crRNA1	−77.48	−75.1	2.38
hs‐crRNA2	−77.77	−75.46	2.31
hs‐crRNA3	−77.76	−76.16	1.6
hs‐crRNA4	−77.96	−76.65	1.31
hs‐crRNA5	−78.48	−76.94	1.54

### 2.4 Hs‐crRNAs Increase the Specificity of CRISPR/Cas13a System

**Figure 3:**
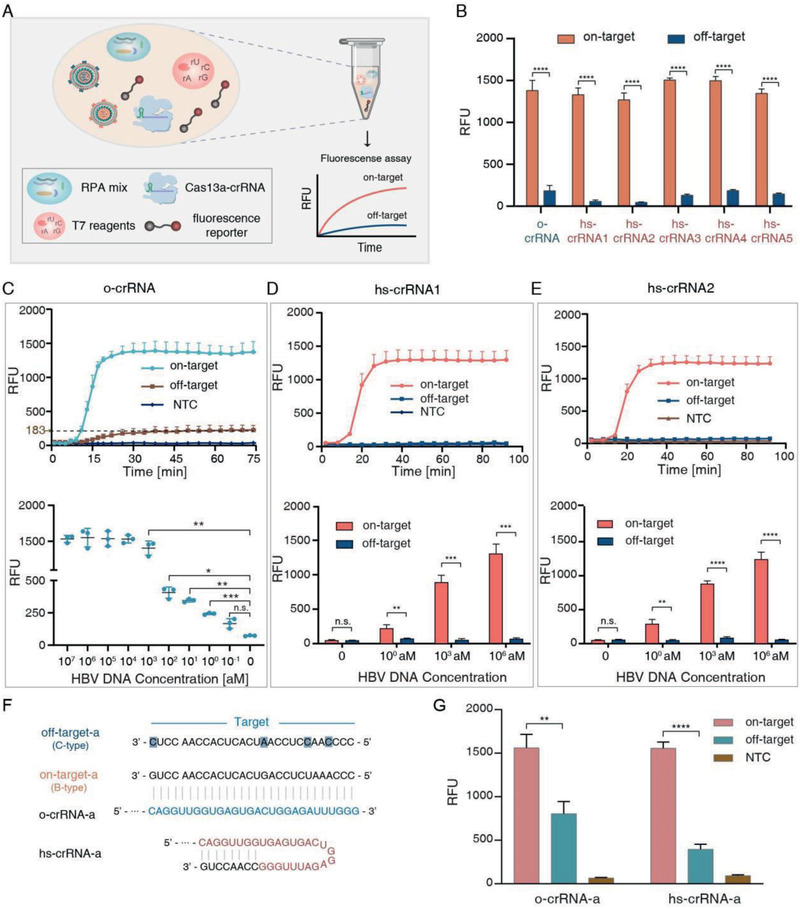
Hairpin‐spacer crRNAs increase the SNPs identification ability of CRISPR/Cas13a system. A) Workflow of HBV genotyping platform based on CRISPR/Cas13a system. All reagents were encapsulated in one pellet. B) End‐point fluorescence intensity for on‐target and off‐target HBV using o‐crRNA and hs‐crRNAs. C) Kinetics of fluorescence assay for on‐target and off‐target HBV using o‐crRNA (up) and the sensitivity results of Cas13a/o‐crRNA (bottom). D) The specificity (up) and sensitivity (bottom) of Cas13a system using hs‐crRNA1. E) The specificity (up) and sensitivity (bottom) of Cas13a system using hs‐crRNA2. F) Target sequence and design of o‐crRNA and hs‐crRNA. The SNP‐induced mismatch for off‐target (C‐type) HBV is highlighted in navy blue. G) End‐point fluorescence intensity for on‐target and off‐target using o‐crRNA and hs‐crRNA. *n* = 3 replicates; two‐tailed Student's *t*‐test; bars represent mean ± S.D. *, *P* < 0.05; **, *P* < 0.01; ***, *P* < 0.001; ****, *P* < 0.0001. n.s., not significant.

To investigate the relationship between Δ*G* and the specificity of Cas13a system, we applied ordinary crRNA and hairpin‐spacer crRNAs for HBV genotyping. The workflow of HBV genotyping is shown in **Figure** **3**A. All reagents, including RPA mix, T7 transcription mix, and Cas13a nuclease assay reagents, were encapsulated in a single pellet. The whole detection process takes less than 2 h at 37 °C. At first, we tested the specificity of the hs‐CRISPR/Cas13a system as well as ordinary CRISPR/Cas13a system for HBV genotyping by utilizing hs‐crRNAs and o‐crRNA, respectively. As shown in **Table** **2** and Figure 3B, the on‐target signals for Cas13a/hs‐crRNA1, Cas13a/hs‐crRNA2 to Cas13a/hs‐crRNA5 (1297.28 ± 137.77, 1235.58 ± 105.31, 1505.02 ± 23.71, 1498.09 ± 50.15, and 1346.29 ± 53.31 RFU, respectively) were approximate to Cas13a/o‐crRNA (1380 ± 120.12 RFU). The on‐target results were consistent with our observations based on Gibbs free energy analysis that the Cas13a protein binding with o‐crRNA and hs‐crRNAs possessed an overall high‐level affinity to on‐target RNA.

**Table 2:** Experimental data of various crRNAs used in this study

crRNAs	Free energy of crRNA‐spacer [kJ mol^−1^]	End‐point fluorescence intensity		Discrimination factor (DF)
		F.I. _[on]_	F.I. _[off]_	
o‐crRNA	−3.68	1380 ± 120.12	183.67 ± 63.53	8.03 ± 2.26
hs‐crRNA1	−68.20	1297.28 ± 137.77	58.2 ± 16.65	23.1 ± 4.32
hs‐crRNA2	−74.48	1235.58 ± 105.31	45 ± 6.56	27.63 ± 1.87
hs‐crRNA3	−43.5136	1505.02 ± 23.71	130.09 ± 13.8	11.65 ± 1.1
hs‐crRNA4	−23.012	1498.09 ± 50.15	185.01 ± 13.73	8.11 ± 0.35
hs‐crRNA5	−111.7128	1346.29 ± 53.31	145.01 ± 13.2	9.32 ± 0.53
o‐crRNA‐a	−12.9704	1561 ± 154.21	804.67 ± 139.75	1.96 ± 0.2
hs‐crRNA‐a	−58.576	1555.86 ± 72.07	395.6 ± 59.08	3.98 ± 0.49

The discrimination factor (DF) of Cas13a/crRNA duplex is determined by Equation (6)

(6) $$\mathrm{DF}\;=\;\frac{F.I._{\left[\mathrm{on}\right]}}{F.I._{\left[\mathrm{off}\right]}}$$

where F.I._[on]_ and F.I._[off]_ mean the end‐point fluorescence intensity of on‐target and off‐target signals, respectively. As shown in Table 2, the DF values of hs‐crRNA1 and hs‐crRNA2 (27.63 ± 1.87 and 23.1 ± 4.32, respectively) were about threefold higher than o‐crRNA (8.03 ± 2.26). Whereas the DF values of hs‐crRNA3 (11.65 ± 1.1), hs‐crRNA4(8.11 ± 0.35), and hs‐crRNA5 (9.32 ± 0.53) were about 1.5‐fold, 1‐fold, and 1.2‐fold higher compared to o‐crRNA (8.03 ± 2.26), respectively. Our data suggested that off‐target signal of Cas13a was successfully suppressed by introducing extra secondary structures in crRNA. The difference in DF values for all crRNAs indicated that Δ*G* values of crRNAs were positively related to the specificity of the Cas13a protein. According to our experimental results of all hs‐crRNAs to on‐target and off‐target HBV, we propose a hairpin structure design strategy to enhance the specificity of the hs‐CRISPR/Cas13a system. By comparing the results of hs‐crRNA1 and hs‐crRNA2 with hs‐crRNA3, we concluded that the SNP‐induced mismatch should be contained in the stem structure of hs‐crRNA to efficiently enhance the Cas13a's specificity (Figure 3D,E; Figure S6, Supporting Information). According to the results of hs‐crRNA1 (10‐bp stem), hs‐crRNA4 (5‐bp stem), and hs‐crRNA5 (15‐bp stem), the stem length of hairpin structure should be set at a proper range which was also determined by the location of the stem and the target sequence. For our experiments, the optimal stem lengths were 8‐bp to 10‐bp.

Since hs‐crRNA1 and hs‐crRNA2 possessed the best performance in HBV genotyping, we further explored the sensitivity of hs‐crRNA1 and hs‐crRNA2 as well as o‐crRNA. The limits of detection (LOD) of o‐crRNA, hs‐crRNA1, and hs‐crRNA2 were all 10^0^ × 10^−18^ m (100 copies mL^−1^) (Figure 3C–E). These results manifested that hairpin‐spacer crRNAs improved the specificity of Cas13a without sacrificing the sensitivity. Moreover, we analyzed the standard curves of the sensitivity of o‐crRNA as well as hs‐crRNA1 and hs‐crRNA2 using the dose–response regression (Figure S7, Supporting Information). To investigate the performance of the Cas13a/hs‐crRNA duplex in complex sample, we mixed HBV target DNA with five kinds of food‐sourced bacteria gDNA and tested the specificity of the hs‐CRISPR/Cas13a system. The amounts of bacteria gDNA were all 10 ng. According to the results provided in Figure S8 of the Supporting Information, both the Cas13a/hs‐crRNA1 and Cas13a/hs‐crRNA2 showed high‐specificity in the complex sample, demonstrating the stability of the hs‐CRISPR/Cas13a system.

To validate the feasibility of the hs‐CRISPR/Cas13a system, we selected another 28‐nt sequence containing four random SNPs for HBV B‐type and C‐type as the target of the o‐crRNA‐a and hs‐crRNA‐a. As shown in Figure 3F, hs‐crRNA‐a was obtained by extending the 3′‐end of o‐crRNA‐a to form a 10‐bp stem at the repeat‐proximal region. We examined the specificity of both o‐crRNA‐a and hs‐crRNA‐a for HBV genotyping by single‐pellet detection and the results are provided in Figure 3G and Figure S9 (Supporting Information). The on‐target signals of o‐crRNA‐a and hs‐crRNA‐a were 1561 ± 154.21 RFU, and 1555.86 ± 72.07 RFU, respectively, whereas the off‐target signals were 804.67 ± 139.75 and 395.6 ± 59.08 RFU, respectively. The DF values of o‐crRNA‐a and hs‐crRNA‐a were calculated and displayed in Table 2. Notably, the hs‐crRNA‐a increased the discrimination factor of the Cas13a protein by two‐fold compared to o‐crRNA‐a, demonstrating the versatility of hairpin‐spacer crRNAs to enhance the specificity of the CRISPR/Cas13a system.

### 2.5 Molecular Docking Simulations of Cas13a and hs‐crRNA2

**Figure 4:**
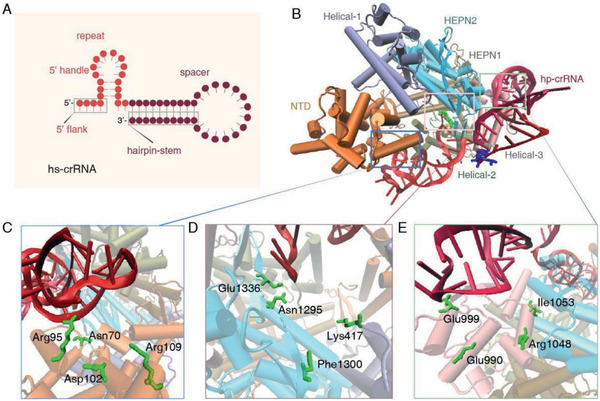
Molecular docking simulations of Cas13a and hs‐crRNA2. A) Structure of hs‐crRNA2. The repeat and spacer of hs‐crRNA2 are shown in light red and dark red, respectively. B) Cartoon representation of Cas13a–crRNA duplex. The 5′ flank and the 3′ flank of Cas13a protein are shown as green and blue licorice, respectively. C,D,E) Mode of specific interactions between hs‐crRNA2 and C) NTD, D) Helical‐1 and HEPN‐2, and E) Helical‐3, respectively. The main interaction for hs‐crRNA stabilization on the Cas13a is electrostatic. However, Arg95, Glu1336, and Glu990 of Cas13a protein interact with hs‐crRNA through the hydrogen bond.

Since hairpin‐spacer crRNAs can increase the identification ability of Cas13a system to SNPs, we further performed the molecular docking simulations to illustrate the binding conformation of Cas13a protein with hs‐crRNA2 and target RNA. Protein Data Bank ID 5W1H was selected for Cas13a. The structure of hs‐crRNA2 is provided in **Figure** **4**A. In the Cas13a/hs‐crRNA binary system, the hs‐crRNA is located in the groove of the REC lobe (NTD) and the NUC lobe (Helical‐2, Helical‐3, and HEPN‐2) of Cas13a protein, as shown in Figure 4B. As shown in Table S2 of the Supporting Information, the interaction between Cas13a and hs‐crRNA2 was mainly electrostatics due to Δ*H* ^0^ < 0 and Δ*S* ^0^ > 0. The 5′ handle of hs‐crRNA localizes on the NTD surface (Figure 4C), close to channel formed between the NTD and the Helical‐1 domains. According to previous reports,^[^ ^9^, ^23^, ^24^ ^]^ crRNA overspreading on the surface of the NTD domain is helpful for subsequent catalysis processes. The 5′ flank is located in the groove of the Helical‐1 and the HEPN‐2 domains (Figure 4D). The 3′ flank is oriented to the interface groove of the Helical‐2 and the Helical‐3 domains, closing to the Helical‐3 domain. The 3′ handle of hs‐crRNA localizes on the Helical‐3 surface (Figure 4E). These domains trapped the hs‐crRNA as clamps, and Cas13a protein is accessible for nuclease activity via conformational changes.

### 2.6 Molecular Docking Study of Cas13a/hs‐crRNA2 Duplex and Target RNA

**Figure 5:**
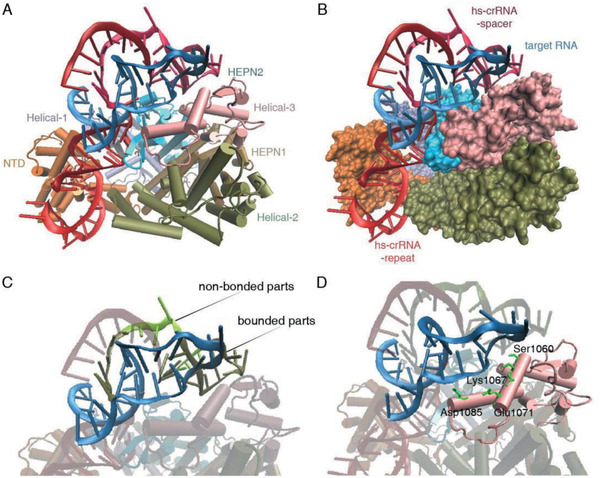
Molecular docking study of Cas13a/hs‐crRNA2 and target RNA. A,B) Two views of the Cas13a/hs‐crRNA2/target RNA complex are shown as cartoon (A) and surface (B) representations. The hs‐crRNA2‐repeat, hs‐crRNA2‐spacer, and target RNA are shown as light red, dark red, and blue cartoons, respectively. C) Mode of specific interactions between the spacer sequence of hs‐crRNA2 and target RNA. The bonded and nonbonded parts of the spacer sequence are shown as cartoons in tan and green, respectively. Hs‐crRNA2 interacts with target RNA through hydrophobic interaction, hydrogen bond, and base stacking. D) Mode of specific interactions between the Helical‐3 domain and target RNA. Target RNA binding to the Helical‐3 is governed by electrostatic force.

In the ternary system, the docking calculations indicated that the recognition of target RNA was guided by the crRNA spacer for Cas13a/hs‐crRNA2 duplex (**Figure** **5**A,B). During RNA recognition of the Cas13a/hs‐crRNA2 duplex, the primary force was hydrophobic and the secondary forces are hydrogen bond, electrostatic, and base stacking. The spacer of hs‐crRNA interacted with target RNA by 37–42, 47–58, and 62–64 NTs, counting from the 5′ end (Figure 5C). In addition, the hydrogen bond and base stacking were the secondary contributing forces. Besides, target RNA bond to Cas13a via interaction with the Helical‐3 domain (Figure 5D). The interaction between target RNA and the Helical‐3 domain was governed by electrostatic force. The Helical‐3 domain was close to the HEPN‐2 domain, and target RNA recognition facilitated the formation of HEPN domain that decided the nuclease activity of Cas13a protein. Our calculations were consistent with previous reports studying the mechanisms of Cas13a activity ^[^ ^9^, ^23^, ^24^ ^]^. These results suggest that hairpin‐spacer crRNAs can be applied to Cas13a system as an effective strategy to promote the specificity.

## 3 Conclusion

In this study, we presented a strategy termed hs‐CRISPR to increase the specificity of CRISPR/Cas13a system. Three main enhancements were developed and achieved. First, hairpin‐spacer crRNAs with engineered secondary structures were proposed to suppress the nonspecific activity of CRISPR/Cas13a system. CRISPR/Cas technology is reported to show high off‐target effects which impedes its applications and there lacks a rational guideline for high‐specificity Cas13a–crRNA design. Herein, we developed crRNAs with hairpin‐spacer and evaluated the binding energetics of Cas13a protein and RNA by fluorescence quenching mechanism. Our enhanced system showed a high on‐target affinity of Cas13a while suppressed the off‐target activity compared to ordinary crRNA. Second, we established an HBV genotyping platform to evaluate the feasibility of hs‐CRISPR to promote the specificity of CRISPR/Cas13a system. Our platform possessed a high sensitivity of 1 × 10^−18^ m (100 copies mL^−1^) and hairpin‐spacer crRNAs increased the discrimination ability of Cas13a system by at least three‐fold than ordinary crRNA. The whole assay takes about 1 h and can be encapsulated in a single pellet which is time‐saving and easy to operate. Third, we performed theoretical simulations to illustrate the mechanisms of Cas13a nuclease activity by interacting with hairpin‐spacer crRNA and target RNA. Our findings suggest that hairpin‐spacer crRNAs are compatible with traditional Cas13a system.

In summary, this enhanced hs‐CRISPR system was verified to increase the specificity of CRISPR/Cas13a system both theoretically and experimentally in this study. By comparison of ordinary crRNAs and hairpin‐spacer crRNAs, the off‐target nuclease activity of Cas13a could be suppressed by introducing an extra secondary structure to decrease the binding affinity to off‐target RNA. Furthermore, genotyping platforms based on the enhanced hs‐CRISPR system possessed several advantages such as fast, easy‐operation and programmability. Collectively, our enhanced hs‐CRISPR system will facilitate future point‐of‐care molecular diagnostics and RNA interference based on CRSIPR/Cas13a technology.

## 4 Experimental Section

##### Materials and Instruments

All DNA templates and primers were synthesized from Sangon Biotechnology Co., Ltd. (Shanghai, China). The fluorophore quencher‐labeled RNA reporter was purchased from Takara Biotechnology Co., Ltd. (Dalian, China). For nucleic acid targets, plasmids containing HBV genome of genotype B and C were provided by Prof. Y. M. Wen (Fudan University, Shanghai, China). All sequences and plasmids used in this study were provided in Table S1 of the Supporting Information. The LwaCas13a protein was expressed and obtained from GenScript Co., Ltd. (Jiangsu, China). RPA was performed following instruction of TwistAmp Basic kit from TwistDx Inc. (Cambridge, UK). The dsDNAs were purified using MinElute gel extraction kit from Qiagen (Shanghai, China). crRNAs were purified using RNAXP clean beads from Beckman Coulter (Shanghai, China). The HiScribe T7 Quick High Yield RNA Synthesis kit, murine RNase inhibitor, T7 RNA polymerase, and NTP mix buffer were purchased from New England Biolabs (Beijing, China). MEGAclear Transcription Clean‐up kit and RNase‐free water were ordered from Thermo Fisher (Shanghai, China).

The real‐time fluorescence intensity of nuclease assay was determined by a microplate reader (BioTek Synergy H1). Electrophoresis was carried out using Tanon EPS‐100 electrophoresis system. Fluorescence of Cas13a protein was measured using Agilent Technologies Cary Eclipse Fluorescence Spectrometer.

##### Preparation of crRNAs

The designed crRNA with T7 promoter sequence was used for DNA template and annealed with a short T7 primer (5′‐TAATACGACTCACTATAGGG‐3′) in 1× annealing buffer containing 100 × 10^−3^ m Tris‐HCl (pH 7.5), 10 × 10^−3^ m EDTA, 1 m NaCl. In vitro transcription of crRNA was performed using the HiScribe T7 Quick High Yield RNA Synthesis kit overnight at 37 °C. Transcription reactions contained NTP buffer mix (10 × 10^−3^ m each NTP final), T7 RNA polymerase mix, template DNA (2 µg), murine RNase inhibitor (1 U µL^−1^ final). After the synthesis of crRNA, adequate RNase‐free water, and DNase I (4 units final) were added into the system to digest the DNA template by incubating at 37 °C for 15 min. The transcribed crRNAs were then purified with twofold volume of RNAXP clean beads and subsequently stored at −20 °C. The obtained crRNAs were measured by Nanodrop and analyzed by denaturing urea polyacrylamide gel electrophoresis (urea/PAGE).

##### HBV RNA Preparation and Purification

First, HBV plasmids were amplified by RPA using TwistAmp Basic kit. RPA primers for HBV targets were designed using NCBI Primer – Blast. Notably, the HBV RPA forward primers should contain a short T7 sequence at its 5′‐end for later transcription. Second, the amplified products were evaluated by agarose gel electrophoresis and then purified by MinElute PCR purification kit (Qiagen, China). Third, the purified HBV DNA amplicons contained extended T7 sequences were transcribed into RNA using the HiScribe T7 Quick High Yield RNA Synthesis kit overnight at 37 °C. Finally, the transcribed HBV RNA were purified by the MEGAclear Transcription Clean‐up kit and then evaluated by urea/PAGE.

##### Fluorescence Measurement

LwaCas13a proteins have natural fluorophores (tyrosine and tryptophan residues) and allow the study of conformational changes. Intrinsic fluorescence spectra of Cas13a protein (5 × 10^−6^ m) incubated with various concentrations of crRNAs (0 × 10^−6^ to 2.5 × 10^−6^ m) were measured using Agilent Technologies Cary Eclipse Fluorescence Spectrometer in cuvette. Afterward, the Cas13a/crRNA duplex was incubated with transcribed HBV RNA (concentrations ranging from 0 × 10^−6^ to 1 × 10^−6^ m) and emission spectra were recorded. The excitation and emission wavelengths were set at 285 and 295–500 nm, respectively. Experiments were performed at 25 and 37 °C for all RNAs.

##### Single Pellet Detection

The reactions of RPA‐HBV DNA amplification, T7 RNA polymerase‐in vitro transcription and Cas13a nuclease assay were integrated in one pellet. Briefly, a 25 µL of single‐pellet reaction solution consisted of 0.48 × 10^−6^ m forward primer, 0.48 × 10^−6^ m reverse primer, 1× RPA buffer, varying amounts of target DNA input, 1 U µL^−1^ murine RNase inhibitor, 2 × 10^−6^ m each rNTP, 1 µL T7 polymerase mix (New England Biolabs), 45 × 10^−9^ m LwCas13a protein, 22.5 × 10^−9^ m crRNA, 250 × 10^−9^ m quenched fluorescent RNA reporter, 5 × 10^−3^ m MgCl_2_, and 14 × 10^−3^ m MgAc. Reactions were proceeded for 2 h at 37 °C in a microplate reader for fluorescence kinetics measurement every 2 min.

##### Molecular Docking Study of Cas13a/crRNA and Cas13a/crRNA/Target RNA Complex

The molecular docking simulation was performed to predict the consensus pose corresponds to the interaction interface at the primary state in binary and ternary systems of Cas13a/crRNA and Cas13a/crRNA‐target RNA, respectively. The docking calculations were processed by the HDOCK server (http://hdock.phys.hust.edu.cn/).^[^ ^40^, ^41^ ^]^ The 3D structure of Cas13a protein was obtained from the Protein Data Bank ID 5W1H. The 3D structures of crRNA and target RNA were generated by the RNAComposer server (http://rnacomposer.cs.put.poznan.pl/).^[^ ^42^ ^]^ Rigid docking calculations were carried out to investigate the binding activity between Cas13a and crRNA, followed for the Cas13a/crRNA duplex and target RNA. Besides, possible interactions were evaluated at the primary state in binary and ternary systems. The docking results were analyzed using Visual Molecular Dynamics.^[^ ^43^ ^]^ The positions of molecules were investigated based on the energy score. The best pose of interaction was selected based on the minimum energy HDOCK score.

##### Statistics Analysis

Statistical analysis was performed by two‐tailed Student's *t*‐test using Graphpad Prism 8.0 and Origin 2021. Each experiment was repeated three times. The results were presented as mean ± S.D.

## Conflict of Interest

The authors declare no conflict of interest.

## Supporting information

Supporting Information

## References

[1] X. Zuo , C. Fan , H.‐Y. Chen , Nat. Biomed. Eng. 2017, 1, 0091. 10.1038/s41551-017-0128-3 PMC5711465 29204310

[2] R. Bruch , G. A. Urban , C. Dincer , Trends Biotechnol. 2019, 37, 791. 31078316 10.1016/j.tibtech.2019.04.005

[3] M. Hu , C. Yuan , T. Tian , X. Wang , J. Sun , E. Xiong , X. Zhou , J. Am. Chem. Soc. 2020, 142, 7506. 32223241 10.1021/jacs.0c00217

[4] X. Jing , B. Xie , L. Chen , N. Zhang , Y. Jiang , H. Qin , H. Wang , P. Hao , S. Yang , X. Li , Nucleic Acids Res. 2018, 46, e90. 29860393 10.1093/nar/gky433 PMC6125684

[5] Z. Ali , A. Mahas , M. Mahfouz , Trends Plant Sci. 2018, 23, 374. 29605099 10.1016/j.tplants.2018.03.003

[6] Q. Wang , X. Liu , J. Zhou , C. Yang , G. Wang , Y. Tan , Y. Wu , S. Zhang , K. Yi , C. Kang , Adv. Sci. 2019, 6, 1901299. 10.1002/advs.201901299 PMC6794629 31637166

[7] S. Riesenberg , M. Chintalapati , D. Macak , P. Kanis , T. Maricic , S. Pääbo , Nucleic Acids Res. 2019, 47, e116. 31392986 10.1093/nar/gkz669 PMC6821318

[8] A. V. Anzalone , L. W. Koblan , D. R. Liu , Nat. Biotechnol. 2020, 38, 824. 32572269 10.1038/s41587-020-0561-9

[9] L. Liu , X. Li , J. Ma , Z. Li , L. You , J. Wang , M. Wang , X. Zhang , Y. Wang , Cell 2017, 170, 714. 28757251 10.1016/j.cell.2017.06.050

[10] O. O. Abudayyeh , J. S. Gootenberg , S. Konermann , J. Joung , I. M. Slaymaker , D. B. T. Cox , S. Shmakov , K. S. Makarova , E. Semenova , L. Minakhin , K. Severinov , A. Regev , E. S. Lander , E. V. Koonin , F. Zhang , Science 2016, 353, aaf5573. 27256883 10.1126/science.aaf5573 PMC5127784

[11] A. East‐Seletsky , M. R. O'Connell , S. C. Knight , D. Burstein , J. H. D. Cate , R. Tjian , J. A. Doudna , Nature 2016, 538, 270. 27669025 10.1038/nature19802 PMC5576363

[12] S. Shmakov , O. O. Abudayyeh , K. S. Makarova , Y. I. Wolf , J. S. Gootenberg , E. Semenova , L. Minakhin , J. Joung , S. Konermann , K. Severinov , F. Zhang , E. V. Koonin , Mol. Cell 2015, 60, 385. 26593719 10.1016/j.molcel.2015.10.008 PMC4660269

[13] J. S. Gootenberg , O. O. Abudayyeh , J. W. Lee , P. Essletzbichler , A. J. Dy , J. Joung , V. Verdine , N. Donghia , N. M. Daringer , C. A. Freije , C. Myhrvold , R. P. Bhattacharyya , J. Livny , A. Regev , E. V. Koonin , D. T. Hung , P. C. Sabeti , J. J. Collins , F. Zhang , Science 2017, 356, 438. 28408723 10.1126/science.aam9321 PMC5526198

[14] J. Quan , C. Langelier , A. Kuchta , J. Batson , N. Teyssier , A. Lyden , S. Caldera , A. McGeever , B. Dimitrov , R. King , J. Wilheim , M. Murphy , L. P. Ares , K. A. Travisano , R. Sit , R. Amato , D. R. Mumbengegwi , J. L. Smith , A. Bennett , R. Gosling , P. M. Mourani , C. S. Calfee , N. F. Neff , E. D. Chow , P. S. Kim , B. Greenhouse , J. L. DeRisi , E. D. Crawford , Nucleic Acids Res. 2019, 47, e83. 31114866 10.1093/nar/gkz418 PMC6698650

[15] Y. Shan , X. Zhou , R. Huang , D. Xing , Anal. Chem. 2019, 91, 5278. 30873832 10.1021/acs.analchem.9b00073

[16] B. Wienert , S. K. Wyman , C. D. Richardson , C. D. Yeh , P. Akcakaya , M. J. Porritt , M. Morlock , J. T. Vu , K. R. Kazane , H. L. Watry , L. M. Judge , B. R. Conklin , M. Maresca , J. E. Corn , Mol. Biol. 2018, 364, 286. 10.1126/science.aav9023 PMC6589096 31000663

[17] L. Zhang , H. T. Rube , C. A. Vakulskas , M. A. Behlke , H. J. Bussemaker , M. A. Pufall , Nucleic Acids Res. 2020, 48, 5037. 32315032 10.1093/nar/gkaa231 PMC7229833

[18] S. Q. Tsai , N. Wyvekens , C. Khayter , J. A. Foden , V. Thapar , D. Reyon , M. J. Goodwin , M. J. Aryee , J. K. Joung , Nat. Biotechnol. 2014, 32, 569. 24770325 10.1038/nbt.2908 PMC4090141

[19] F. A. Ran , P. D. Hsu , C.‐Y. Lin , J. S. Gootenberg , S. Konermann , A. E. Trevino , D. A. Scott , A. Inoue , S. Matoba , Y. Zhang , F. Zhang , Cell 2013, 154, 1380. 23992846 10.1016/j.cell.2013.08.021 PMC3856256

[20] T. Zhou , R. Huang , M. Huang , J. Shen , Y. Shan , D. Xing , Adv. Sci. 2020, 7, 1903661. 10.1002/advs.201903661 PMC7341088 32670752

[21] C. Myhrvold , C. A. Freije , J. S. Gootenberg , O. O. Abudayyeh , H. C. Metsky , A. F. Durbin , M. J. Kellner , A. L. Tan , L. M. Paul , L. A. Parham , K. F. Garcia , K. G. Barnes , B. Chak , A. Mondini , M. L. Nogueira , S. Isern , S. F. Michael , I. Lorenzana , N. L. Yozwiak , B. L. MacInnis , I. Bosch , L. Gehrke , F. Zhang , P. C. Sabeti , Science 2018, 360, 444. 29700266 10.1126/science.aas8836 PMC6197056

[22] S. Wang , H. Huang , J. Liu , L. Wei , L. Wu , W. Xiong , P. Yin , T. Tian , X. Zhou , Adv. Sci. 2020, 7, 1903770. 10.1002/advs.201903770 PMC7341091 32670753

[23] M. R. O'Connell , J. Mol. Biol. 2019, 431, 66. 29940185 10.1016/j.jmb.2018.06.029

[24] G. J. Knott , A. East‐Seletsky , J. C. Cofsky , J. M. Holton , E. Charles , M. R. O'Connell , J. A. Doudna , Nat. Struct. Mol. Biol. 2017, 24, 825. 28892041 10.1038/nsmb.3466 PMC5961731

[25] J. S. Gootenberg , O. O. Abudayyeh , M. J. Kellner , J. Joung , J. J. Collins , F. Zhang , Science 2018, 360, 439. 29449508 10.1126/science.aaq0179 PMC5961727

[26] J. Zhang , C. Li , X. Zhi , G. A. Ramón , Y. Liu , C. Zhang , F. Pan , D. Cui , Anal. Chem. 2016, 88, 1294. 26675240 10.1021/acs.analchem.5b03729

[27] P. Sheng , K. A. Flood , M. Xie , Front. Bioeng. Biotechnol. 2020, 8, 8. 32850763 10.3389/fbioe.2020.00940 PMC7427337

[28] D. Zhong , K. Yang , Y Wang , X. Yang , Talanta 2017, 175, 217. 28841982 10.1016/j.talanta.2017.07.035

[29] J. Liu , Y. Zhang , H. Xie , L. Zhao , L. Zheng , H. Ye , Small 2019, 15, 1902989. 10.1002/smll.201902989 31523917

[30] R. Abolhasan , A. Mehdizadeh , M. R. Rashidi , L. A. Maleki , M. Yousefi , Biosens. Bioelectron. 2019, 129, 164. 30708263 10.1016/j.bios.2019.01.008

[31] X. Qi , X. Liu , L. Matiski , R. Rodriguez Del Villar , T. Yip , F. Zhang , S. Sokalingam , S. Jiang , L. Liu , H. Yan , Y. Chang , ACS Nano 2020, 14, 4727. 32275389 10.1021/acsnano.0c00602

[32] D. Han , X. Qi , C. Myhrvold , B. Wang , M. Dai , S. Jiang , M. Bates , Y. Liu , B. An , F. Zhang , H. Yan , P. Yin , Science 2017, 358, eaao2648. 29242318 10.1126/science.aao2648 PMC6384012

[33] D. D. Kocak , E. A. Josephs , V. Bhandarkar , S. S. Adkar , J. B. Kwon , C. A. Gersbach , Nat. Biotechnol. 2019, 37, 657. 30988504 10.1038/s41587-019-0095-1 PMC6626619

[34] M. Fekri Noodeh , A. Divsalar , A. Seyedarabi , A. A. Saboury , J. Mol. Liq. 2018, 249, 265.

[35] B. Ghalandari , K. Asadollahi , A. Shakerizadeh , A. Komeili , G. Riazi , S. K. Kamrava , N. Attaran , J. Photochem. Photobiol., B 2019, 192, 131. 30735954 10.1016/j.jphotobiol.2019.01.012

[36] B. Ghalandari , A. Divsalar , A. A. Saboury , T. Haertlé , K. Parivar , R. Bazl , M. Eslami‐Moghadam , M. Amanlou , Spectrochim. Acta, Part A 2014, 118, 1038. 10.1016/j.saa.2013.09.126 24161866

[37] R. L. Joseph , Principles of Fluorescence Spectroscopy, Springer, Boston, MA 2006.

[38] S. Zolghadri , A. A. Saboury , A. Golestani , A. Divsalar , S. Rezaei‐Zarchi , A. A. Moosavi‐Movahedi , J. Nanopart. Res. 2009, 11, 1751.

[39] A. Dadras , G. H. Riazi , A. Afrasiabi , A. Naghshineh , B. Ghalandari , F. Mokhtari , J. Biol. Inorg. Chem. 2013, 18, 357. 23397429 10.1007/s00775-013-0980-x

[40] Y. Yan , H. Tao , J. He , S.‐Y. Huang , Nat. Protoc. 2020, 15, 1829. 32269383 10.1038/s41596-020-0312-x

[41] Y. Yan , D. Zhang , P. Zhou , B. Li , S.‐Y. Huang , Nucleic Acids Res. 2017, 45, W365. 28521030 10.1093/nar/gkx407 PMC5793843

[42] M. Popenda , M. Szachniuk , M. Antczak , K. J. Purzycka , P. Lukasiak , N. Bartol , J. Blazewicz , R. W. Adamiak , Nucleic Acids Res. 2012, 40, e112. 22539264 10.1093/nar/gks339 PMC3413140

[43] W. Humphrey , A. Dalke , K. Schulten , J. Mol. Graphics 1996, 14, 33. 10.1016/0263-7855(96)00018-5 8744570

