# Supplementary material for: Hairpin‐Spacer crRNA‐Enhanced CRISPR/Cas13a System Promotes the Specificity of Single Nucleotide Polymorphism (SNP) Identification
Source: Adv Sci (Weinh). 2021 Jan 31;8(6):2003611. doi: 10.1002/advs.202003611 (PMC7967054; doi:10.1002/advs.202003611)
Supplement: Supplementary file 1 — Supporting Information [file ADVS-8-2003611-s001.pdf]

WILEY-VCH

© 2020 Wiley-VCH GmbH

ADVANCED SCIENCE
Open Access

Supporting Information

for *Adv. Sci.,* DOI: 10.1002/advs.202003611

Hairpin-spacer crRNA Enhanced CRISPR/Cas13a System Promotes the Specificity of Single Nucleotide Polymorphisms (SNPs) Identification

Yuqing Ke, Shiyi Huang, Behafarid Ghalandari, Sijie Li, Antony R. Warden, Jingqi Dang, Lin Kang, Yu Zhang, Yunqing Wang, Yiqing Sun, Jinglin Wang, Daxiang Cui, Xiao Zhi, Xianting Ding**

Supporting Information

Hairpin-spacer crRNA enhanced CRISPR/Cas13a system promotes the specificity of single nucleotide polymorphisms (SNPs) identification

Yuqing Ke, Shiyi Huang, Behafarid Ghalandari, Sijie Li, Antony R. Warden, Jingqi Dang, Lin Kang, Yu Zhang, Yunqing Wang, Yiqing Sun, Jinglin Wang, Daxiang Cui, Xiao Zhi, Xianting Ding**

Table S1. *Sequences of nucleotides used in this study*

RPA Primers used in this study

Name	Sequence
RPA Forward Primer	AATTCTAATACGACTCACTATAGGGACTCGTGGTGGACTTCTCTCAATTTTCTAGGG
RPA Reverse Primer	CCAACAAGAAGATGAGGCATAGCAGCAGGATG

crRNA sequences used in this study

Name	Complete crRNA sequence	Spacer Sequence
o-crRNA	5'-GAUUUAGACUACCCCAAAAACGAAGGGGACUAAAAC UAACGAGGACAAAUUGGAGGACAACAGG-3'	UAACGAGGACAAAUUGGAGGACAAC AGG
hs-crRNA1	5'-GAUUUAGACUACCCCAAAAACGAAGGGGACUAAAAC UAACGAGGACAAAUUGGAGGACAACAGGAACACCUGUUGUC C-3'	UAACGAGGACAAAUUGGAGGACAAC AGGAACACCUGUUGUCC
hs-crRNA2	5'-GAUUUAGACUACCCCAAAAACGAAGGGGACUAAAAC UAACGAGGACAAAUUGGAGGACAACAGGCCUCGUUA-3'	UAACGAGGACAAAUUGGAGGACAAC AGGACAACAGGCCUCGUUA
hs-crRNA3	5'-GAUUUAGACUACCCCAAAAACGAAGGGGACUAAAAC UAACGAGGACAAAUUGGAGGACAACAGGUCCAAUUUGU-3'	UAACGAGGACAAAUUGGAGGACAAC AGGUCCAAUUUGU
hs-crRNA4	5'-GAUUUAGACUACCCCAAAAACGAAGGGGACUAAAAC UAACGAGGACAAAUUGGAGGACAACAGGAACACCUGU-3'	UAACGAGGACAAAUUGGAGGACAAC AGGAACACCUGU
hs-crRNA5	5'-GAUUUAGACUACCCCAAAAACGAAGGGGACUAAAAC UAACGAGGACAAAUUGGAGGACAACAGGAACACCUGUUGUC CUCCAA-3'	UAACGAGGACAAAUUGGAGGACAAC AGGAACACCUGUUGUCCUCCAA
o-crRNA-a	5'-GAUUUAGACUACCCCAAAAACGAAGGGGACUAAAAC CAGGUUGGUGAGUGACUGGAGAUUUGGG-3'	CAGGUUGGUGAGUGACUGGAGAUU UGGG

hs-crRNA-a	5'-GAUUUAGACUACCCCAAAAACGAAGGGGACUAAAAC CAGGUUGGUGAGUGACUGGAGAUUUGGG CCAACCUG-3'	CAGGUUGGUGAGUGACUGGAGAUU UGGG CCAACCUG

HBV plasmids used in this study

Name	GenBank ID
HBV B-genotype	AF100309
HBV C-genotype	AF461363

Table S2. Binding results of Cas13a interacting with crRNA at 37 °C

Group	ΔH^0 [kJ mol^{-1}]	ΔS^0 [kJ K^{-1} mol^{-1}]	$\Delta G^0{}_1$ [kJ mol^{-1}]
o-crRNA	-13.27	83.92	-39.31
hs-crRNA1	-48.68	-36.16	-37.46
hs-crRNA2	-13.28	78.56	-37.64
hs-crRNA3	8.85	159.29	-38.64
hs-crRNA4	42.78	263.14	-38.83
hs-crRNA5	2.95	135.67	-39.12

Table S3. Thermodynamic parameters of Cas13a/crRNA interacting with target RNA at 37 °C

Group	Target	ΔH^0 [kJ mol^{-1}]	ΔS^0 [kJ K^{-1} mol^{-1}]	$\Delta G^0{}_2$ [kJ mol^{-1}]
o-crRNA	on	14.75	173.53	-39.06
	off	-14.75	73.81	-37.64
hs-crRNA1	on	42.78	266.96	-40.02
	off	14.01	166.84	-37.74
hs-crRNA2	on	19.18	191.24	-40.13
	off	-14.75	74.38	-37.82
hs-crRNA3	on	-23.6	50.5	-39.12
	off	-32.45	16.34	-37.52
hs-crRNA4	on	-11.8	88.11	-39.13
	off	-17.7	64.86	-37.82
hs-crRNA5	on	-8.85	98.38	-39.36
	off	-20.65	55.35	-37.82

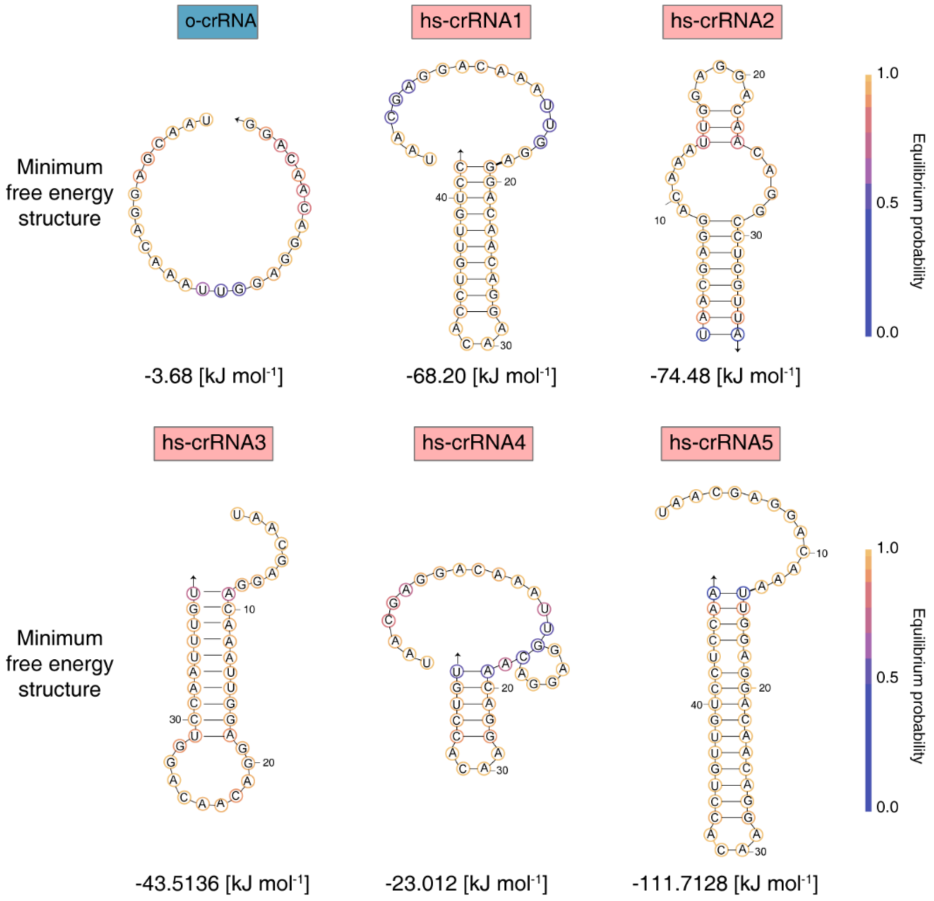

Figure S1. Predicted minimum free energy structures of the crRNA-spacers of o-crRNA, hs-crRNA1, hs-crRNA2, hs-crRNA3, hs-crRNA4, and hs-crRNA5.

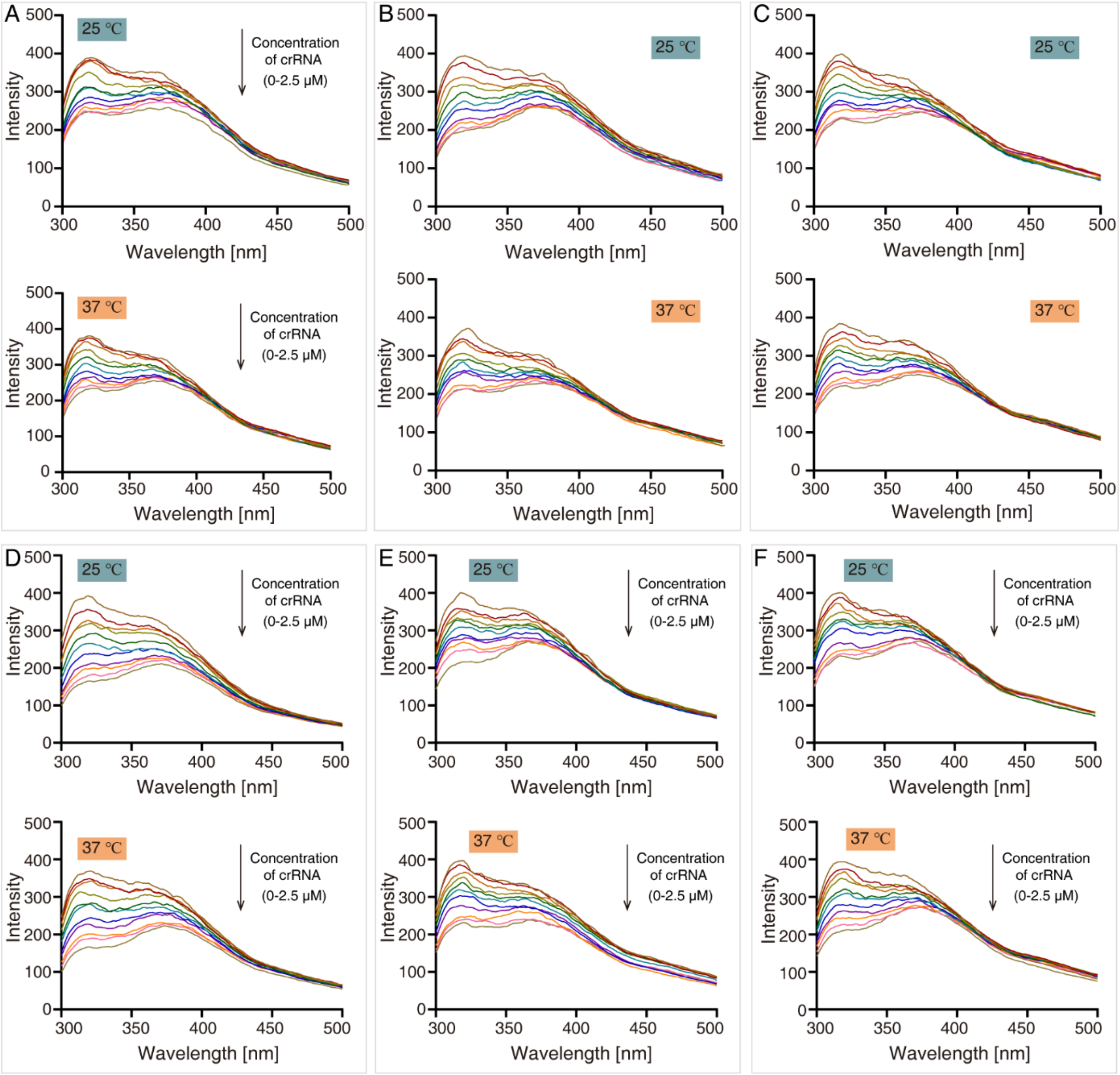

Figure S2. Fluorescence spectra of Cas13a interacting with crRNA at 25 °C and 37 °C. (A) o-crRNA, (B) hs-crRNA1, (C) hs-crRNA2, (D) hs-crRNA3, (E) hs-crRNA4, (E) hs-crRNA5.

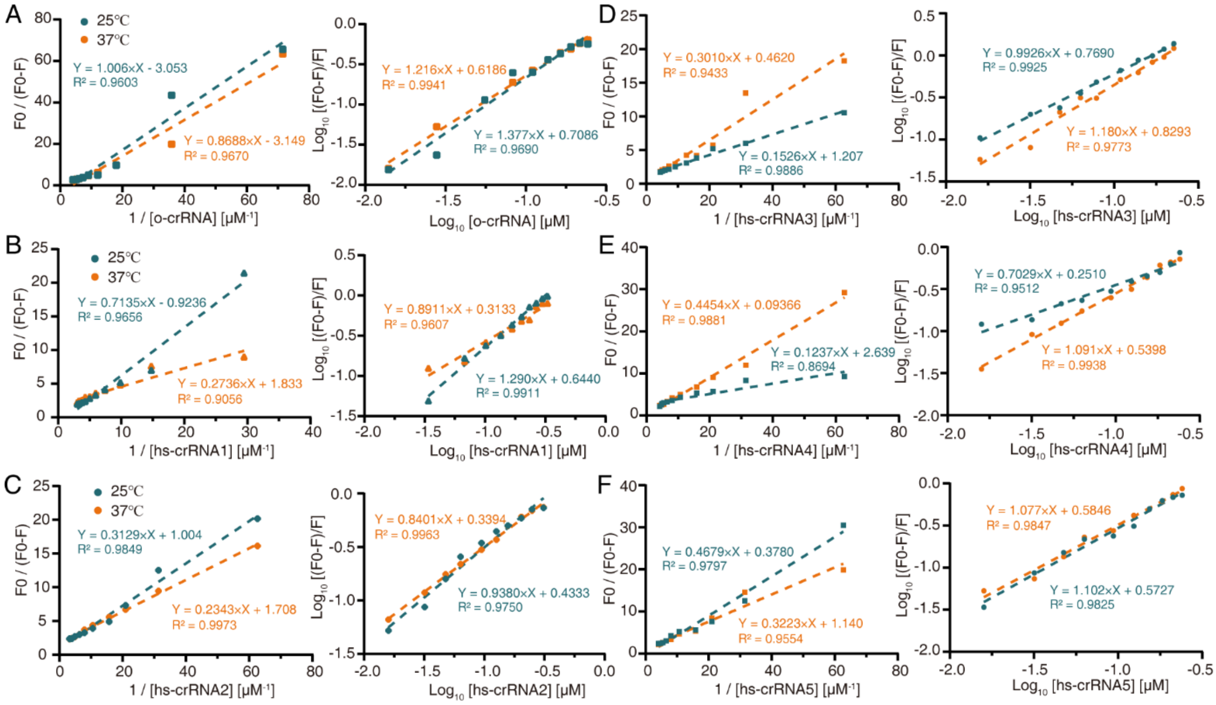

Figure S3. Stern-Volmer plots of Cas13a interacting with crRNA at 25 and 37°C. (A) o-crRNA, (B) hs-crRNA1, (C) hs-crRNA2, (D) hs-crRNA3, (E) hs-crRNA4, (E) hs-crRNA5.

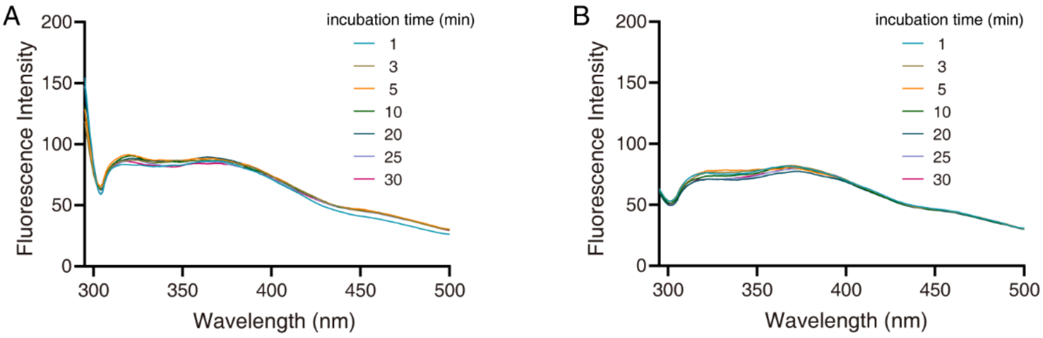

Figure S4. The fluorescence spectra of Cas13a protein interacting with o-crRNA (A) and hs-crRNA1 (B) under different incubation time (0-30 min) at 37 ℃. Lines with different colors represent various incubation time.

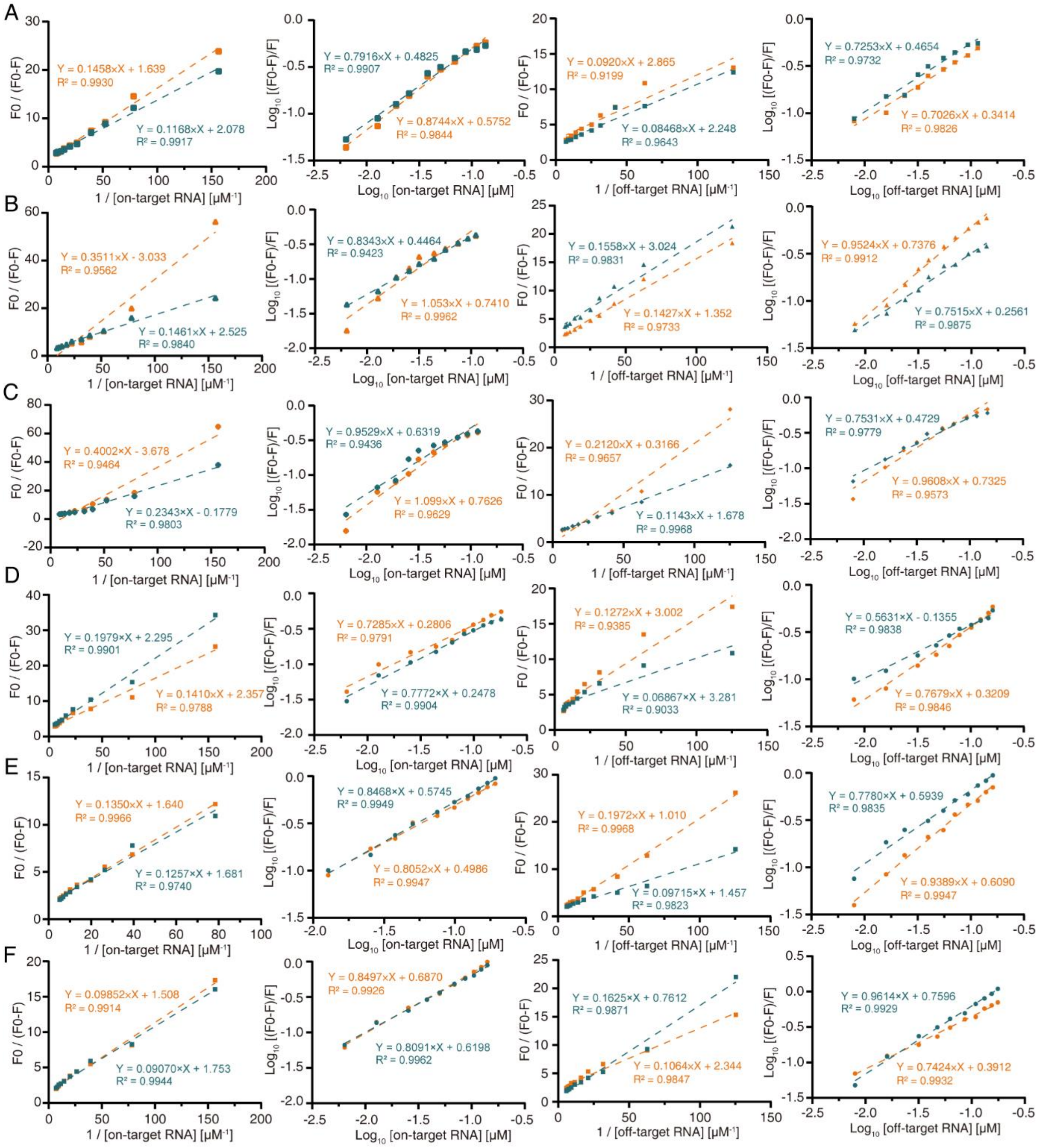

Figure S5. Stern-Volmer plots of Cas13a-crRNA duplex interacting with target RNA. (A) o-crRNA. (B) hs-crRNA1. (C) hs-crRNA2. (D) hs-crRNA3, (E) hs-crRNA4, (E) hs-crRNA5.

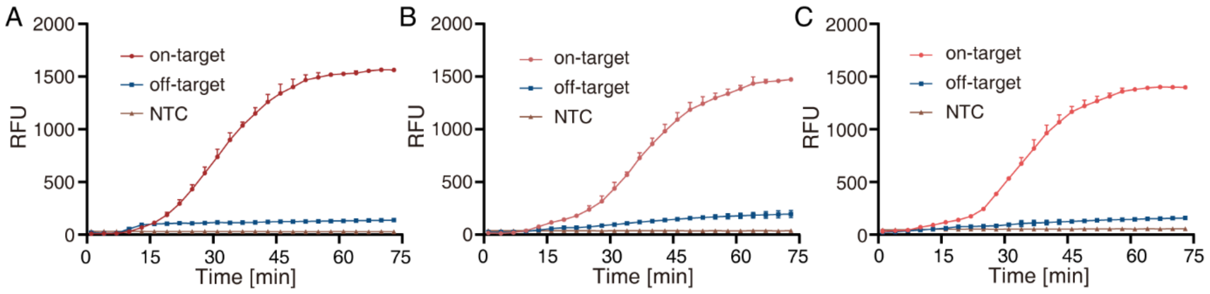

Figure S6. Kinetics of fluorescence assay for on-target and off-target HBV using (A) hs-crRNA3, (B) hs-crRNA4, and (C) hs-crRNA5. n=3 replicates; bars represent mean ± S.D.

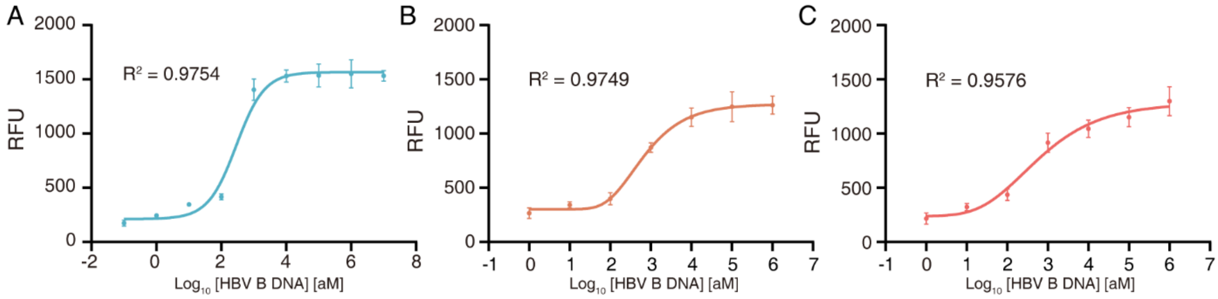

Figure S7. Standard curves of the detection sensitivity using (A) Cas13a/o-crRNA, (B) Cas13a/hs-crRNA1, and (C) Cas13a/hs-crRNA2. n=3 replicates; bars represent mean ± S.D.

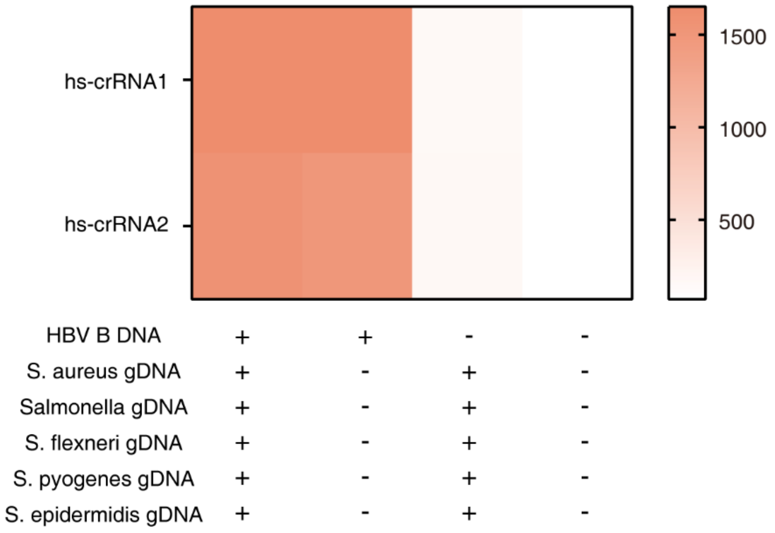

Figure S8. Detection performance of Cas13a/hs-crRNA1, and Cas13a/hs-crRNA2 in complex sample. S. aureus, S. flexneri, S. pyogenes and S. epidermidis represent Staphylococcus aureus, Shigella flexneri, Streptococcus pyogenes, and Staphylococcus epidermidis, respectively.

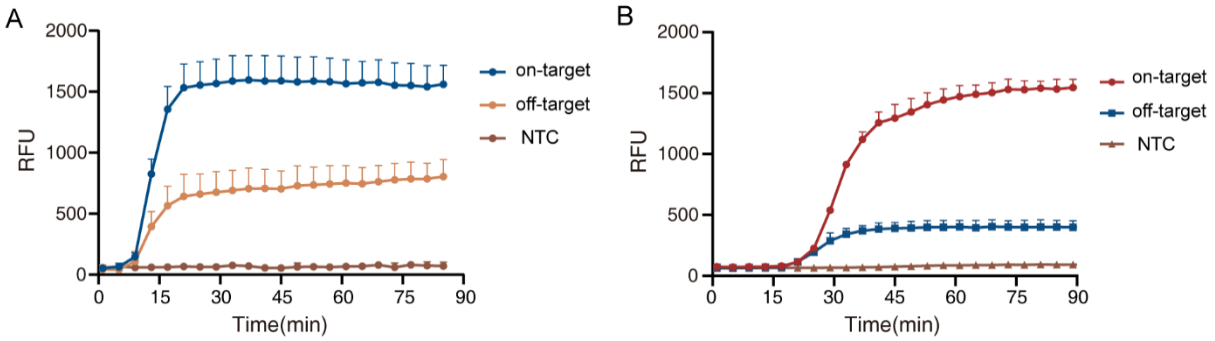

Figure S9. Kinetics of fluorescence assay for on-target and off-target HBV using (A) o-crRNA-a, (B) hs-crRNA-a. n=3 replicates; bars represent mean ± S.D.
